# Method for High-Yield Hydrothermal Growth of Silica Shells on Nanoparticles

**DOI:** 10.3390/ma14216646

**Published:** 2021-11-04

**Authors:** Max Willinger, Martin Felhofer, Erik Reimhult, Ronald Zirbs

**Affiliations:** Department of Nanobiotechnology, University of Natural Resources and Life Sciences, Muthgasse 11, A-1190 Vienna, Austria; max.willinger@boku.ac.at (M.W.); felhofer_martin@groupwise.boku.ac.at (M.F.); erik.reimhult@boku.ac.at (E.R.)

**Keywords:** hydrothermal synthesis, coating, silica, carbon nanotubes, polystyrene, surfactant-assisted

## Abstract

Coating processes are commonly used in materials science to protect a core or modify material properties. We describe a hydrothermal coating process using TEOS (tetraethyl orthosilicate), a widely used precursor for silica coatings, on three representative template materials (carbon nanotubes, silica, and polystyrene nanoparticles) with different properties and shapes. We compare the efficiency of previously published protocols for silica coatings at room temperature and atmospheric pressure with the hydrothermal process at 160 °C and 3 bar. The hydrothermal method achieves higher yields and thicker silica coatings with the same amount of precursor when compared to the conventional way, thus offering higher effectiveness. Furthermore, the hydrothermal coating process yields more homogeneous shells with a higher density, making hydrothermal coating the method of choice when mechanical integrity and low permeability of the coating are required.

## 1. Introduction

One of the essential operations in materials science is coating materials with a thin layer of functional material. These coatings are applied to protect a sensitive core material or, more commonly, to bridge properties between the core material and the surrounding bulk material. A functional and well-designed coating improves solubility and dispersibility of particles in a bulk polymer or solution and increases storage stability by protecting the core against exposure to pH, oxygen, light, or unwanted physisorption at the surface.

Another innovative application of coating processes is to produce differently shaped hollow structures by removing the core material after the coating process [1,2,3,4] Hollow mesoporous silica structures are used as highly lightweight aerogels and in medical and cosmetic formulations. For example, they can encapsulate active ingredients or bind or absorb molecules, e.g., hollow silica spheres supplying lipids into the skin in daily facial creams [5,6,7].

The following parameters are critical for the coating process: (i) type of template (reactive groups of the template used), (ii) chemical compatibility of core and shell, (iii) solvent, and (iv) temperature. The most frequently described method for coating different core templates with silica and other oxide shells is using well-soluble precursors at room temperature [8,9,10,11,12]. Tissot et al. used this method to coat latex particles with silica shells [13,14]. Nandiyanto et al. successfully coated silica on polystyrene particles with the same method [15,16].

For coating processes with poorly soluble or sol-gel incompatible (e.g., metal alkoxide) precursors, the hydrothermal method at temperatures above 100 °C and high pressures is used [17]. For example, Song et al. applied the hydrothermal coating process to coat magnesium hydroxide onto the surface of an Mg–2Zn–Mn–Ca–Ce alloy as a protective layer against corrosion [18]. Titrici et al. used the hydrothermal approach to synthesize metal oxide hollow spheres using carbohydrate and metal salt mixtures [19]. Additionally, Y. Zhang et al. used the hydrothermal method for the electrochemical application of mesoporous vanadyl hydroxide [20].

In addition to improving the solubility of precursors, hydrothermal or solvothermal processes can be used to supersaturate solutions. By controlled ramping of the temperature beyond the supersaturation temperature for a high concentration of precursor, nucleation and crystal growth are enormously favored. Hence, such methods have found extensive use in the synthesis of, e.g., monodisperse crystalline oxide nanomaterials. We can expect similar benefits for coating materials by heterogeneous nucleation and growth from template nanomaterials [21,22,23].

Tetraethyl orthosilicate (TEOS) is a hydrophobic precursor, which enables growing silica shells on different templates. Polystyrene and silica particles are typical spherical templates due to their synthetic tunability and surface chemistry [24,25]. However, TEOS also allows the coating of nanostructures with an aspect ratio greater than one. For example, carbon nanotubes were successfully coated with silica using this approach [26,27,28].

**Figure 1:**
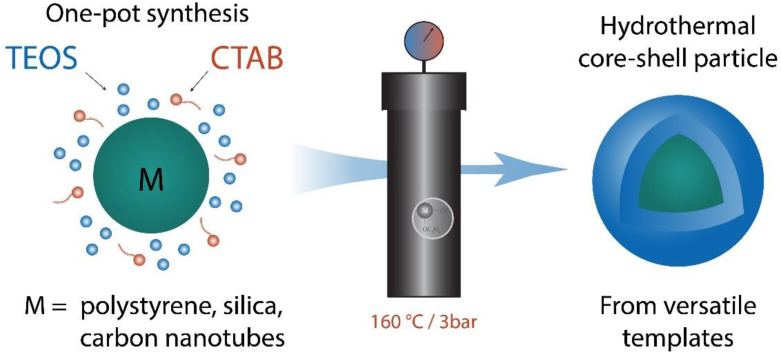
Schematic illustration of the one-pot synthesis procedure for silica shells. TEOS on polystyrene, silica, and carbon nanotube nanoparticles.

The hydrothermal process allows for growing coatings with sol-gel compatible precursors. Based on the known advantages of solvothermal synthesis methods described above, we assumed that by using this method, higher yields and higher conversion degrees of silica coating using TEOS as hydrophobic precursor materials should be achieved [21,22,23]. Additionally, such a method should apply to core templates of a wide variety of geometries and chemistries. Therefore, we used three representative core types: polystyrene particles (exemplifying organic polymeric cores), silica particles (exemplifying inorganic cores showing a comparable structure as the silica shell), and carbon nanotubes (exemplifying high aspect ratio organic cores). The carbon nanotubes were modified and oxidized before the coating to achieve a homogeneous dispersion after sonication [29]. Water was chosen as a cheap and environmentally friendly solvent for all reactions. Hexadecyltrimethylammonium bromide (CTAB) was used as a surfactant to stabilize the nanoparticle dispersions and control the precursor to (template-)surface area. We directly compared the growth and properties of the silica shells formed on the three different core types at room temperature and under hydrothermal conditions (160 °C; 3 bar) (Figure 1).

## 2. Materials and Methods

### 2.1. Materials

Styrene (≥99%), polyvinylpyrrolidone (PVP, MW = 40,000 g mol^−1^), 2,2′-Azobis (2-methyl-propionamidine)dihydrochloride (AIBA, 97%), carbon nanotubes (CNTs, multi-walled), hydrogen peroxide solution (H_2_O_2_, 30% (*w*/*w*)), ammonium hydroxide solution (25%), hexadecyltrimethylammonium bromide (CTAB, ≥98%), and tetraethyl orthosilicate (TEOS, 98%) were purchased from Sigma-Aldrich (St. Louis, MO, USA). Ethanol (≥96%) and a Zitex^®^-PTFE (Teflon; polytetrafluorethylene) filter were obtained from Roth, and Ethanol (≥99.9%) from AustrAlco (Spillern, Austria). All chemicals were used without further purification.

### 2.2. Preparation of Polystyrene Particles

2.0 g PVP was dissolved in 150 mL DI water in a 250 mL flask equipped with a magnetic stirrer. 8.5 g styrene was added to the mixture during the bubbling of nitrogen gas. The mixture was heated to 70 °C, followed by adding 0.2 g AIBA dissolved in 15 mL DI water to initiate the polymerization. After stirring overnight (~16 h), the particles were centrifuged and washed two times with DI water. The product was dried via freeze-drying overnight and analyzed via TEM.

### 2.3. Preparation of Silica Particles

400 mL of ethanol (>99.9%) were mixed with 8 mL DI water and 24 mL ammonium hydroxide solution under a nitrogen gas atmosphere. 12 mL TEOS was added after 5 min of stirring. The particles were allowed to grow overnight (~16 h). The product was obtained through centrifugation and washed three times with ethanol and three times with water, and the particles were freeze-dried overnight. The received white product was analyzed by transmission electron microscopy (TEM).

### 2.4. Modification of Carbon Nanotubes

2.5 g Carbon nanotubes were dispersed in 400 mL Hydrogen peroxide and heated to 130 °C. The mixture was filtrated through a PTFE filter and washed several times with DI water after refluxing for four hours at 130 °C. The modified carbon nanotubes were dried in an oven at 80 °C for two days. CNTs oxidized with this method show 4–5% oxygen content [29]. This oxidation level is low in comparison with other more aggressive methods and insufficient for water dispersion, but sufficient for further modification.

### 2.5. Coating Process

**Figure 2:**
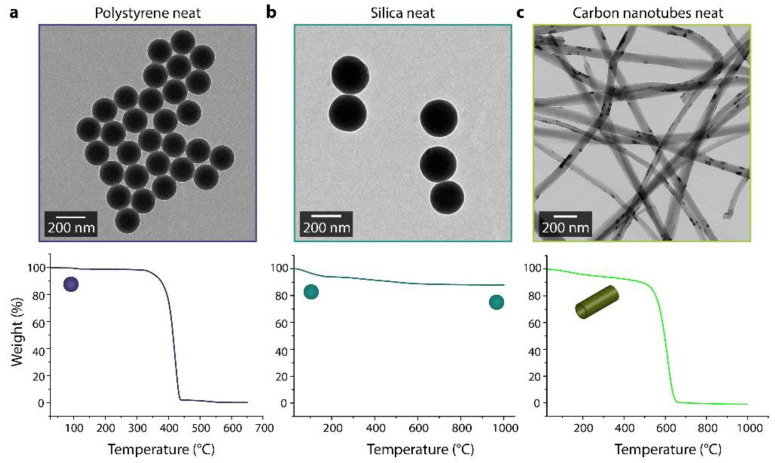
Transmission electron micrographs and thermogravimetric analysis data for the core materials used; (**a**) polystyrene particles (160 ± 5 nm); (**b**) silica nanoparticles (220 ± 11 nm); and (**c**) carbon nanotubes (∅ = 65 nm; l ≈ 5 µm).

Two identical reaction mixtures were prepared. Particles (Table 1 and Figure 2) with a total surface area of 24 m^2^ were dispersed in 40 mL of DI water via sonication. A solution of 0.2 g (0.55 mmol) CTAB dissolved in 5 mL ethanol and 0.4 mL of ammonium hydroxide solution was added under constant magnetic stirring. The mixture continued stirring for 10 min. After a fast addition of the desired amount of TEOS (14, 28, or 42 mmol), one of the mixtures was stirred (~240 rpm) at room temperature overnight (~16 h); the other was stirred in a reactor under hydrothermal conditions, 160 °C and 3 bar, in an oil bath overnight (~16 h). After the completed reaction, the reactor was cooled to room temperature with air while the mixture inside was stirred. Both batches were centrifuged and washed two times with ethanol to remove the unconverted precursor and the excess surfactant and two times with water. The products were freeze-dried overnight and analyzed using TEM, thermogravimetric analysis (TGA), and X-ray photoelectron spectroscopy (XPS).

### 2.6. Methods

Transmission Electron Microscopy (TEM) images of the samples were recorded with an FEI Tecnai G2 (FEI Europe B.V., Vienna, Austria) with 160 kV acceleration voltage on carbon grids. The size distributions of the template particles and the thicknesses of the silica shells were determined manually from >100 particles per sample. Thermal gravimetric analysis (TGA) was performed on a Mettler Toledo TGA/DSC (Mettler Toledo GmbH, Vienna, Austria) with flow rates of 80 mL·min^−1^ synthetic air (reactive gas) and 20 mL·min^−1^ nitrogen gas (protective gas). The temperature range was 25 to 650 °C and 25 to 1000 °C with a heating rate of 10 K·min^−1^. An X-ray photoelectron spectrometer (XPS, Surface Science Instruments, SSX-100 S-probe photoelectron spectrometer, Mountain View, CA, USA) equipped with Al Kα radiation of 200 W was used to collect the XPS data. The Casa XPS software was used for the analysis of the recorded XPS spectra.

#### Raman Spectroscopy

For Raman measurements, the nanoparticle powders were placed on a standard glass slide. A Confocal Raman microscope (CRM) (alpha 300 RA, WITec GmbH, Ulm, Germany) equipped with a piezo motorized scan stage (x-y) was used. The excitation light source was a linearly polarized (0°) coherent compass sapphire green laser λ 532 nm (WITec, Ulm, Germany) focused through a 20× objective (Carl Zeiss, Jena, Germany). The Raman scattering signal was collected by the same objective, delivered by an optic multifiber (∅ = 50 µm) to the spectrometer (600 g·mm^−1^ grating, UHTS 300 WITec, Ulm, Germany) and recorded by an attached CCD camera (Andor DU401ABV, Belfast, North Ireland). The Control Four acquisition software (WITec, Ulm, Germany) was used for control of the measurement. The laser power for all measurements was set to 40 mW and the integration time was 60 s. Project FIVE Plus (WITec, Ulm, Germany) was used for spectral processing and data analysis. The extracted spectra were analyzed with Opus 7.5 software (Bruker, Bremen, Germany).

## 3. Results and Discussion

### 3.1. XPS and Thermogravimetric Analysis Reveal the Growth of Silica and a Thicker and Denser Shell Using the Hydrothermal Method

**Figure 3:**
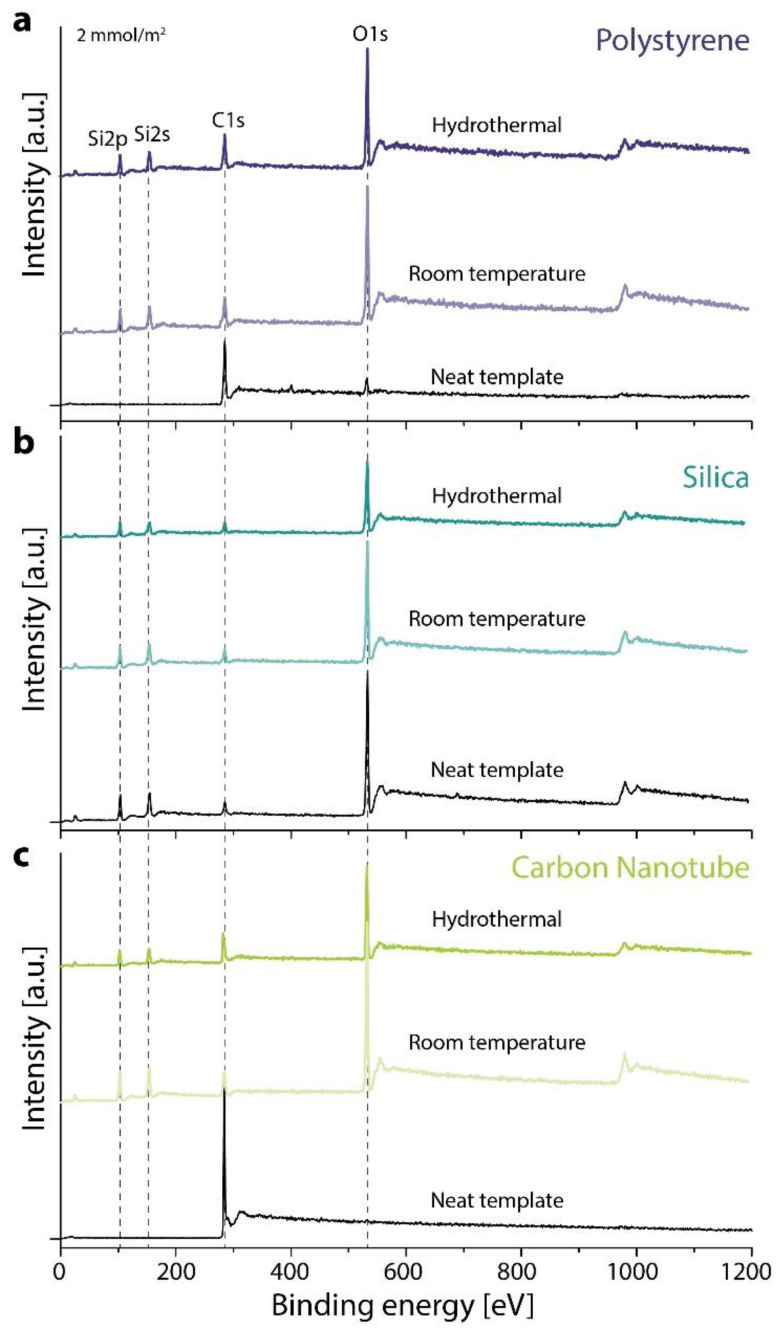
XPS survey spectra of the 2 mmol TEOS per m^2^ samples, (**a**) polystyrene template (**b**) silica template (**c**) carbon nanotube template.

Figure 3 shows the XPS survey spectra measured for the pure template and after silica coating by the standard room temperature and hydrothermal methods. The survey spectra prove the presence of a silica shell for all templates and both methods. The Si 2p peak is observed at 104 eV and the Si 2s at 155 eV as for pure SiO_2_ with no chemical shift for either method [30]. In the survey spectrum, peaks of the pure silica particle template are also visible.

**Figure 4:**
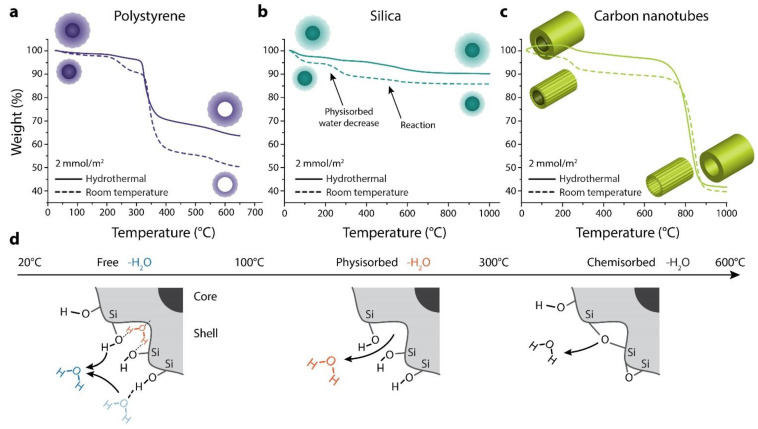
TGA analysis for the 2 mmol TEOS per m^2^ samples. (**a**) polystyrene template, (**b**) silica template, (**c**) carbon nanotube template, dashed line: room temperature method, solid line: hydrothermal method, (**d**) physisorbed and chemisorbed water is released during TGA.

To quantify the mass of the grown silica shell and the thermal decomposition of volatile materials, we used thermogravimetric analysis (TGA). The polystyrene templates showed a significant mass loss in the range of 300 to 400 °C, which resembles the removed core template (Figure 4a). Here, the hydrothermal method led to a lower mass loss when compared to the room temperature method because of the thicker silica shell formed (Figure 4a). As shown in Table 2, the shell thickness can be easily increased from 20 to 55 nm, changing the precursor and method. In contrast, the silica templates show only a decrease in volatile materials because the silica core is stable upon heating (Figure 4b). This decrease is generally due to hydroxyl groups on the surface, physisorbed water, chemical reactions, and other impurities (Figure 4d). Therefore, the TGA diagram shows the evaporation of hydroxyl groups first, followed by physisorbed water, and finally the chemisorbed (reaction) water (arrows in Figure 4b,d).

Furthermore, the lower mass decrease for the hydrothermal process is due to the thicker and denser shell because the surface-to-volume ratio allows fewer hydroxyl groups. To demonstrate the potential use of the hydrothermal method, we selected carbon nanotubes (CNT) as a representative sample to investigate templates with a higher aspect ratio. The TGA profiles exhibit the characteristic mass loss curves for CNTs in the range of 700 to 900 °C (Figure 4c). Surprisingly, the two methods resulted in almost the same mass loss; thus, the same shell thickness would be expected based on the TGA results. However, the shell thickness measured by TEM was lower for the room temperature method (18–24 nm) than the hydrothermal method (24–42 nm) (see Table 2). On the other hand, CNT templates gained a lower shell thickness than the spherical templates (Table 2); this might be due to the lower template diameter (65 nm) and the isotropic porous structure of the CNTs. Still, the hydrothermal method demonstrated an increase in the shell thickness and lower mass loss, especially for spherical templates (Table 2). Therefore, to elucidate the nanostructural differences of the silica shells, particularly of the CNTs, we applied transmission electron microscopy (TEM).

### 3.2. Homogenous and Smooth Shells via Hydrothermal Synthesis

**Figure 5:**
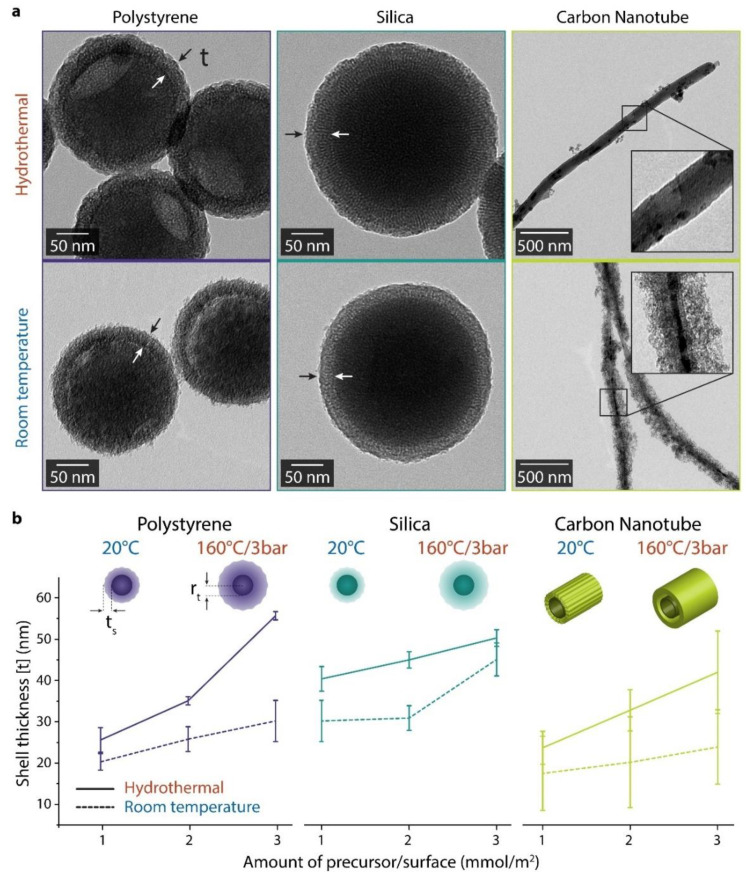
Silica-coated templates show a higher shell thickness and greater conversion efficiency with the hydrothermal method. (**a**) TEM images of silica-coated templates for the 2 mmol TEOS per m^2^ samples. For carbon nanotubes, the hydrothermal treatment produces a smooth surface coating compared to the room temperature method, for which a porous and rough surface is visible. (**b**) Schematics of the changes of the shell thickness. Note that in each case, the hydrothermal method leads to a significantly thicker shell.

The TEM images in Figure 5a show that both the room temperature and hydrothermal methods lead to successful coatings on all the investigated core types. Even the shells on the silica particles are clearly distinguishable due to their lower densities than the core particles. Both methods show an increase in the shell thickness correlating with the precursor to surface area ratio. For those cores where the coating was performed under hydrothermal conditions, a thicker silica shell is observed. Detailed images and high-resolution overview images for the comparison of the different TEOS concentrations concerning the different synthetic pathways are depicted in Figure S1 (polystyrene core), Figure S2 (silica core), and Figure S3 (carbon nanotubes) (Supplementary Material).

For all investigated templates and both methods, an increase in the precursor to surface area ratio leads to an increase in silica content, as evidenced by the TGA results described above. Figure 5b contains a statistical analysis of the TEM images, which shows that increasing the precursor to surface area ratio also increases the shell thickness. For the polystyrene and the CNT templates, the conversion of the precursor binding to the template is significantly higher for all molar ratios when using the hydrothermal process. The conversion rate at low TEOS molarity is close to zero for the traditional room-temperature method, while it is significant for the hydrothermal method. The difference is especially pronounced for the polystyrene particles with almost no shell formed with the room temperature method. In contrast, thick shells were formed on the same cores by applying the hydrothermal method. It thereby showed the most significant difference for the new hydrothermal method compared to the traditional room temperature method.

There is a difference in the shell thicknesses and densities between the polystyrene and silica templates despite their similarities in size, dispersion, and even TEOS conversion rate. The silica quickly forms a relatively thick shell on the silica particles. However, this shell thickness only increases slightly as the precursor ratio increases. In contrast, the shell thickness on polystyrene particles increases rapidly with the precursor ratio, leading to an even higher shell thickness on polystyrene than on silica cores for 3 mmol/m^2^ TEOS to surface area ratio. Hence, in the current data range, the silica shell formed on silica cores by the hydrothermal method mainly densifies, while the shells grown in the same way on polystyrene grow more in thickness than in density. The room temperature method does not show this difference.

For both methods, the carbon nanotubes (CNT) silica coating is significantly less efficient than the polystyrene and silica particles. The silica conversion on the polystyrene and silica particles is almost independent of the precursor to area ratio. However, an inspection of the TEM micrographs (Figure 5a) reveals that the coating on CNTs also is much less homogeneous than on the other particles. Silica growth from TEOS precursors generally produces a particle-like porous growth, morphing into a uniform porous layer as the surface coverage increases. Still, the impression from TEM is that both synthetic pathways lead predominantly to small particles of silica attaching to the CNT, ending in an inhomogeneous coating of the CNTs. Especially using the conventional method at room temperature, the shell comprised of silica particulates appears very amorphous and inhomogeneous. Only at very high concentrations of precursor (>3 mmol/m^2^) do the CNT shells synthesized at room temperature appear as “closed shells.” Hence, CNTs seem to be a bad template for nucleating growth from TEOS despite the surface treatment and careful dispersion procedure. Alternatively, the high curvature of the CNTs might make the inhomogeneities in the TEOS-grown silica coatings more visible in TEM than on the more extensive, lower-curvature templates.

In summary, we observe a denser shell growing on silica particles, a more amorphous almost particulate layer growing on CNTs, and an intermediate case on the polystyrene particles. These observations indicate that despite the common CTAB coating, the different surface properties of the template materials play a role. Heterogeneous nucleation seems favored over homogeneous nucleation and crystal growth for silica templates compared to the CNT and polystyrene templates, which we expected from their different surface properties. More nucleation sites and homogeneously distributed nucleation sites lead to denser silica growth on silica than on CNT templates. For CNTs, we can even suspect from the observed morphology that homogeneous nucleation and crystal growth occur, and these particles attach to the CNT coating to form the less dense and thinner layer we observed compared to on the other templates.

We note by comparing Figure 5b and the values for produced SiO_2_ in Table 2 that the difference in shell thickness between the two tested methods is much less than the difference in TEOS to silica conversion. With a low contrast in thickness and a large difference in the total amount of silica formed in the shell, it is clear that the density of the hydrothermally grown shells is higher than for the shells produced at room temperature. As shown for the CNT templates discussed above, this difference seems exacerbated for templates where the silica growth nucleates poorly. While only 3 mmol/mm^2^ precursor concentrations yield an approximately complete shell on CNTs with the current room temperature method, an entire shell is already formed by the hydrothermal method at 1 mmol/m^2^ (Supplementary Material Figure S3). Hydrothermal methods promote crystal formation. Although our coatings likely are amorphous, it is in line with expectations that they exhibit higher crystallinity than standard RT methods. This translates to larger crystallites and a higher density of coatings grown by the hydrothermal method.

### 3.3. Raman Spectroscopy of the Nanoparticles Reveals Changes in the Thickness of the Grown Shell and the Structure of the Silica Layers

**Figure 6:**
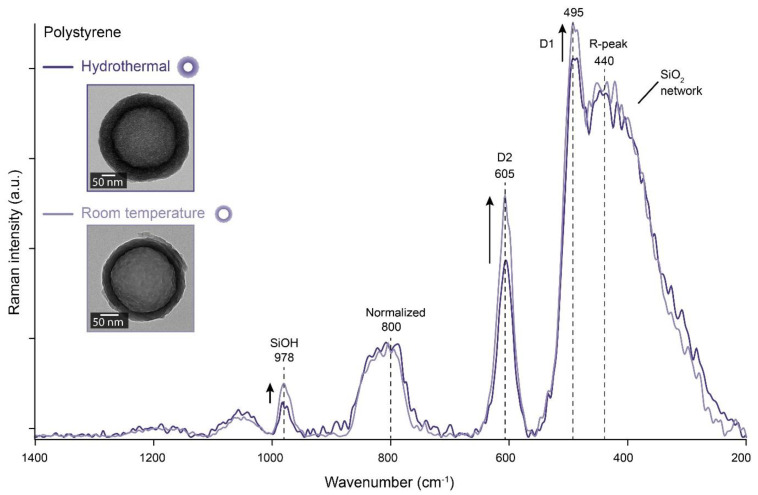
Raman spectroscopy reveals differences in the specific surface of the silica and the relative density of OH groups at the surface between coatings grown by the RT and hydrothermal methods after sintering. Raman spectra of the sintered hollow nanoparticles synthesized on polystyrene templates. Note the increase of the SiOH peak and the D1 and D2.

According to the literature, the Raman spectral features of the SiO_2_ network depend on the particle size and the specific surface [31,32]. To compare the Raman spectra, we must remove the high background contribution caused by the templates (Figures S4–S6). The shells grown on CTAB stabilized polystyrene templates were the most suitable for this comparison as the template can be reliably removed by sintering at 1000 °C. The polystyrene and CTAB template masks all other spectral features before sintering (Figure S4). Removing the CTAB creates mesoporous silica where the CTAB is present [33]. Thus, we sintered the nanoparticles at 1000 °C before the Raman measurements to remove the CTAB, the polystyrene core, and all remaining organic content for the detailed analysis. The obtained spectra were normalized at the 800 cm^−1^ band and compared in Figure 6.

Figure 6 shows the Raman spectra of the nanoparticles synthesized by the hydrothermal and room temperature method (2 mmol/m^2^). The spectra show the characteristic SiO_2_ network bands: SiOH band at 980 cm^−1^, the TO and LO vibrations band at 800 cm^−1^, the D1 band (495 cm^−1^), the D2 band (605 cm^−1^), and the R peak at 440 cm^−1^ (related to the maximum of the distribution of the Si–O–Si angle and therefore to structural features) [31,32]. The D1 band is the vibration mode of SiO_4_ tetrahedra with a non-bridged oxygen atom. The Raman band D2 is assigned to 3-membered rings (SiO)_3_, obtained by surface condensation of two weakly interacting silanols, between 200 and 500 °C [34]. As described in a model of the true 3-cristobalite structure, this condensation generates a (SiO)_3_ ring and a free silanol [34]. Normalized spectra showing differences in the SiOH, D1, and D2 bands can, therefore, demonstrate differences in the surface-to-volume ratio of silica in a sample. Hence, the porosity of the coatings could be compared by Raman spectroscopy.

Comparing the sintered silica coatings grown on polystyrene templates shows stronger SiOH, the D1 and D2 bands (Figure 6) for the shells synthesized at RT than by the hydrothermal method. As these bands are all associated with silica surface structures [32], we conclude that the internal surface area of the silica produced by the RT method is significantly higher than by the hydrothermal method. This can be explained by assuming that the density of the hydrothermally grown silica is higher than for silica synthesized with the RT method. Our observations are made after sintering, but they strongly indicate that the silica crystallites are significantly larger and the voids between them smallest for hydrothermal coatings on polystyrene templates.

## 4. Conclusions

In summary, the universal hydrothermal one-pot method allows the formation of mesoporous shells on different templates, irrespective of shape and size. A key feature of this novel method is that it leads to a significantly higher conversion efficiency than the conventional room-temperature process. This provides a platform for making many designed hollow nanostructures with a lower precursor to surface area ratio. This improvement was most striking on particle templates that are not favorable to silica nucleation and crystal growth, e.g., hydrophobic carbon nanotubes. Our results also indicate that, as expected, the hydrothermal method promotes the growth of denser and less porous silica coatings. Porosity and density are crucial when high mechanical integrity and low permeability are required. We foresee that the presented method will open vast opportunities to precisely control the morphology of nanocomposites for a wide range of applications. Furthermore, due to its easy applicability and comparably high yield, the hydrothermal method can be used as a standard method for synthesizing silica core-shell systems, especially in applications where homogeneous and dense shells are desired.

## Figures and Tables

**Table 1:** Masses and diameters of templates used in this study. ∅ = diameter; l = length of nanotubes.

Core Type	Polystyrene	Silica	Carbon Nanotubes
Dimensions	∅ = 160 ± 5 nm	∅ = 220 ± 11 nm	∅ = 65 nm; l ≈ 5 µm
Mass	0.67 g	2.12 g	0.82 g

**Table 2:** Summary of the SiO_2_ shell thicknesses and relative amounts of SiO_2_ produced for the various coating methods and samples. The shell thickness was determined by TEM measurements on >100 particles per sample.

Template	Method	Amount of Precursor [mmol/m^2^]	Shell Thickness [nm]	Produced SiO_2_ [mmol]	SiO_2_ Fraction [%]
Polystyrene particles ∅ = 160 nm	Room temperature	1	20.3 ± 2.1	0.1 ± <0.1	41.3
	Hydrothermal		25.6 ± 2.8	0.7 ± 0.2	51.3
	Room temperature	2	25.8 ± 3.0	0.2 ± 0.1	50.3
	Hydrothermal		35.1 ± 1.2	1.8 ± 0.4	63.6
	Room temperature	3	30.1 ± 4.9	0.4 ± <0.1	61.6
	Hydrothermal		55.7 ± 0.9	2.3 ± 0.3	67.1
Silica particles ∅ = 220 nm	Room temperature	1	30.2 ± 5.0	0.1 ± <0.1	84.8
	Hydrothermal		40.4 ± 3.2	0.6 ± 0.1	89.6
	Room temperature	2	30.9 ± 3.1	0.8 ± 0.4	85.8
	Hydrothermal		45.0 ± 1.9	1.8 ± 0.3	90.2
	Room temperature	3	45.1 ± 3.8	1.1 ± 0.2	87.2
	Hydrothermal		50.3 ± 2.0	2.3 ± 0.3	91.2
Carbon nanotubes ∅ = 65 nm l = 5 µm	Room temperature	1	17.5 ± 9.4	0.3 ± 0.1	27.7
	Hydrothermal		23.7 ± 3.9	0.5 ± 0.2	29.4
	Room temperature	2	20.2 ± 11.3	0.5 ± 0.1	39.7
	Hydrothermal		32.8 ± 4.7	0.7 ± 0.3	41.6
	Room temperature	3	23.9 ± 9.3	0.6 ± 0.2	50.2
	Hydrothermal		42.0 ± 9.5	1.0 ± 0.6	52.2

## Data Availability

Not applicable.

## Supplementary Materials

The following are available online at https://www.mdpi.com/article/10.3390/ma14216646/s1, Figure S1: TEM images of nanoparticles grown on the polystyrene core template. Shown is the comparison of the hydrothermal (HT) and room temperature (RT) methods for different TEOS concentrations (a) 1 mmol/m^2^, (b) 2 mmol/m^2^, (c) 3 mmol/m^2^, Figure S2: TEM images of the nanoparticles synthetized on the silica core templates. Shown is the comparison of hydrothermal (HT) and room temperature (RT) methods for different TEOS concentrations (a) 1 mmol/m^2^, (b) 2 mmol/m^2^, (c) 3 mmol/m^2^, Figure S3: TEM images the carbon nanotubes bulk rods. Shown is the comparison of the hydrothermal (HT) and room temperature (RT) methods for different TEOS concentrations (a) 1 mmol/m^2^, (b) 2 mmol/m^2^, (c) 3 mmol/m^2^, Figure S4: Raman spectra of the bulk nanoparticles synthesized on polystyrene templates before sintering. The black spectrum shows the neat polystyrene which was used as the core template, Figure S5: Raman spectra of the bulk nanoparticles synthesized on silica templates before sintering. The dark cyan spectrum shows the neat silica that was used as the core template. The orange spectrum is obtained from the CTAB reference compound, Figure S6: Raman spectra of the bulk nanorods synthesized on carbon nanotubes. The black spectrum shows the reference spectrum of the neat carbon nanotubes which were used as the core template.
