# Supplementary material for: Method for High-Yield Hydrothermal Growth of Silica Shells on Nanoparticles
Source: Materials (Basel). 2021 Nov 4;14(21):6646. doi: 10.3390/ma14216646 (PMC8588502; doi:10.3390/ma14216646)
Supplement: Supplementary file 1 [file materials-14-06646-s001.zip › materials-1417736-supplementary.pdf]

materials

Supplementary Materials: Method for High-Yield Hydrothermal Growth of Silica Shells on Nanoparticles

Max Willinger, Martin Felhofer, Erik Reimhult and Ronald Zirbs *

Department of Nanobiotechnology, University of Natural Resources and Life Sciences, Muthgasse 11, A-1190 Vienna, Austria; max.willinger@boku.ac.at (M.W.); felhofer_martin@groupwise.boku.ac.at (M.F.); erik.reimhult@boku.ac.at (E.R.)

* Correspondence: ronald.zirbs@boku.ac.at; Tel.: +43-47654-80206

Citation: Willinger, M.; Felhofer, M.; Reimhult, E.; Zirbs, R. Method for High-Yield Hydrothermal Growth of Silica Shells on Nanoparticles. *Materials* **2021**, *14*, 6646. https://doi.org/10.3390/ma14216646

Academic Editor: Sandra Maria Fernandes Carvalho

Received: 27 September 2021
Accepted: 1 November 2021
Published: 4 November 2021

Publisher's Note: MDPI stays neutral with regard to jurisdictional claims in published maps and institutional affiliations.

Copyright: © 2021 by the authors. Licensee MDPI, Basel, Switzerland. This article is an open access article distributed under the terms and conditions of the Creative Commons Attribution (CC BY) license (http://creativecommons.org/licenses/by/4.0/).

Comparison of the Obtained Nanoparticles of the Two Different Methods and TEOS Concentrations

Figures S1, S2, and S3 show TEM images of the obtained nanoparticles for the hydrothermal (HT) and room temperature (RT) methods for different TEOS concentrations (1, 2 and 3 mmol/m²).

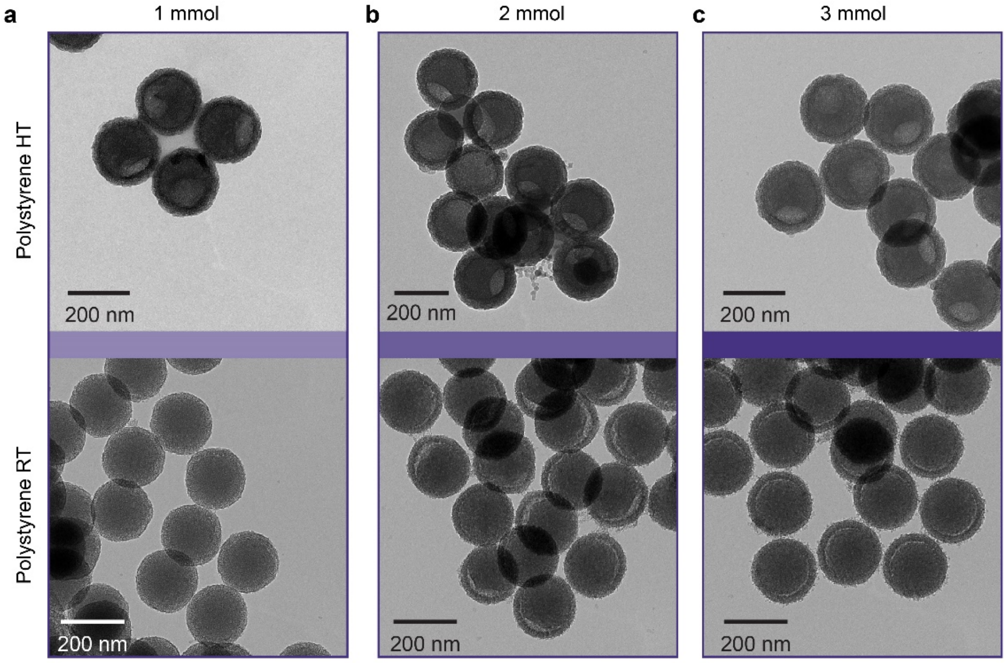

Figure S1. TEM images of nanoparticles grown on the polystyrene core template. Shown is the comparison of the hydrothermal (HT) and room temperature (RT) methods for different TEOS concentrations (**a**) 1 mmol/m², (**b**) 2 mmol/m², (**c**) 3 mmol/m².

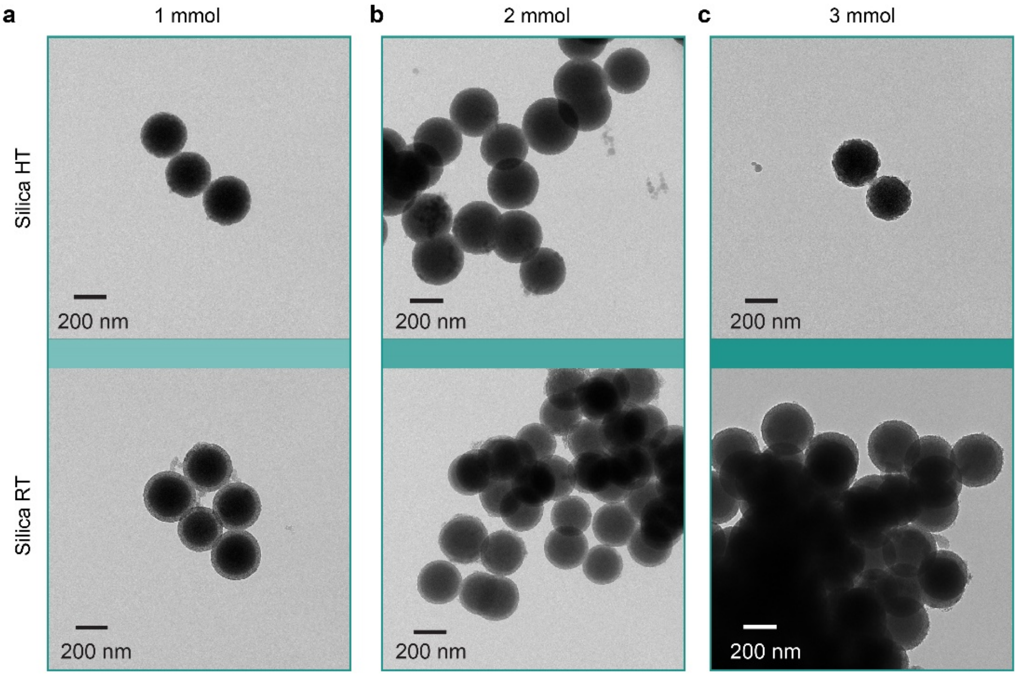

Figure S2. TEM images of the nanoparticles synthetized on the silica core templates. Shown is the comparison of hydrothermal (HT) and room temperature (RT) methods for different TEOS concentrations (**a**) 1 mmol/m², (**b**) 2 mmol/m², (**c**) 3 mmol/m²

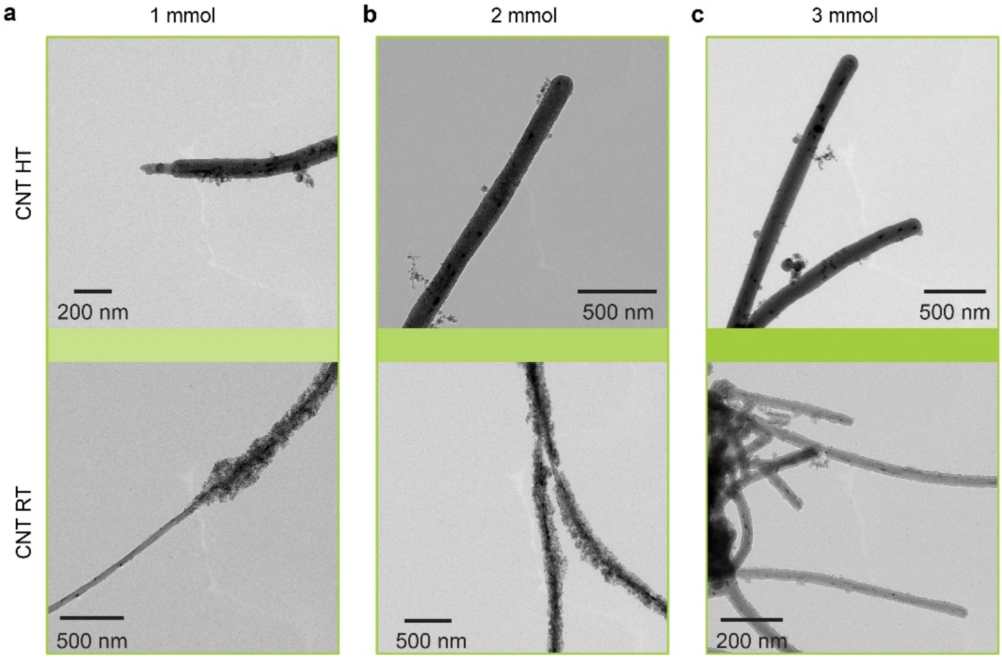

Figure S3. TEM images the carbon nanotubes bulk rods. Shown is the comparison of the hydrothermal (HT) and room temperature (RT) methods for different TEOS concentrations (**a**) 1 mmol/m², (**b**) 2 mmol/m², (**c**) 3 mmol/m²

Additional Raman spectra before sintering

Figure S4 shows clearly that the pure polystyrene spectrum completely masks the signal of the SiO_2 network. Thus, analyzing the spectra regarding the SiO_2 shell is impracticable.

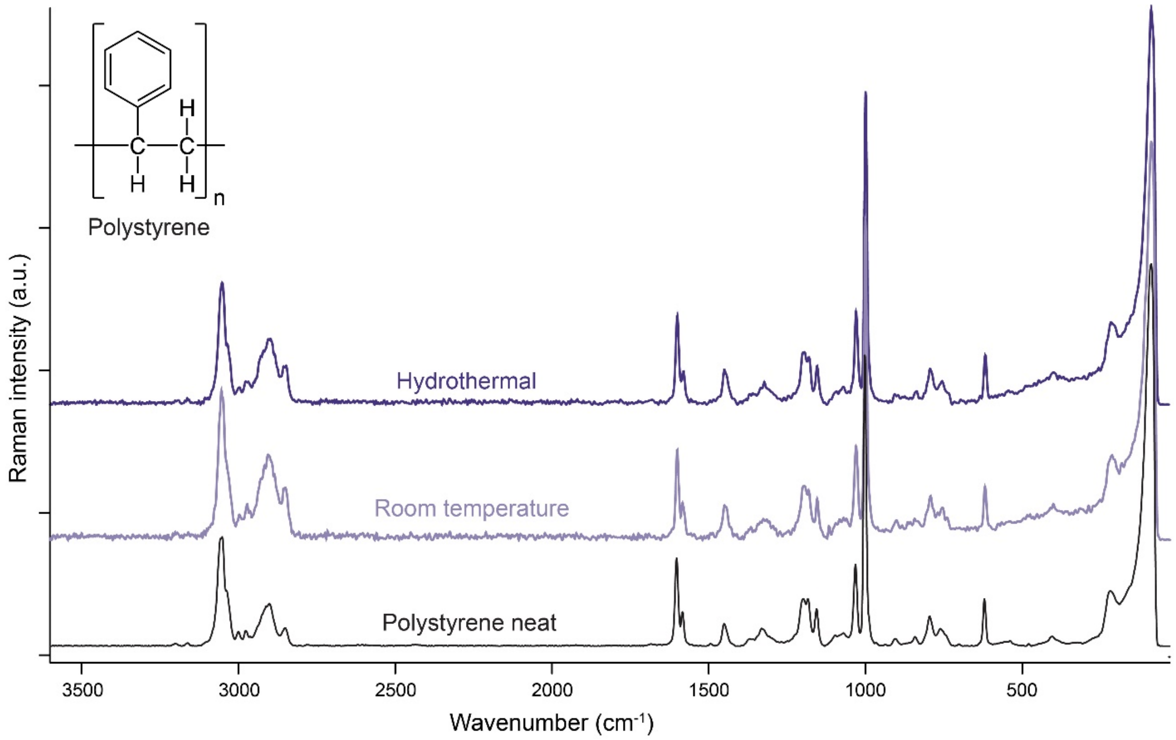

Figure S4. Raman spectra of the bulk nanoparticles synthesized on polystyrene templates before sintering. The black spectrum shows the neat polystyrene which was used as the core template.

The Raman spectra of the bulk silica nanoparticles exhibit a strong spectral contribution of the CTAB surfactant (orange spectrum). The SiO_2 network bands are at 967, 800 cm^{-1}, and the D1 band. Note that the D2 band is absent before sintering (Figure S5).

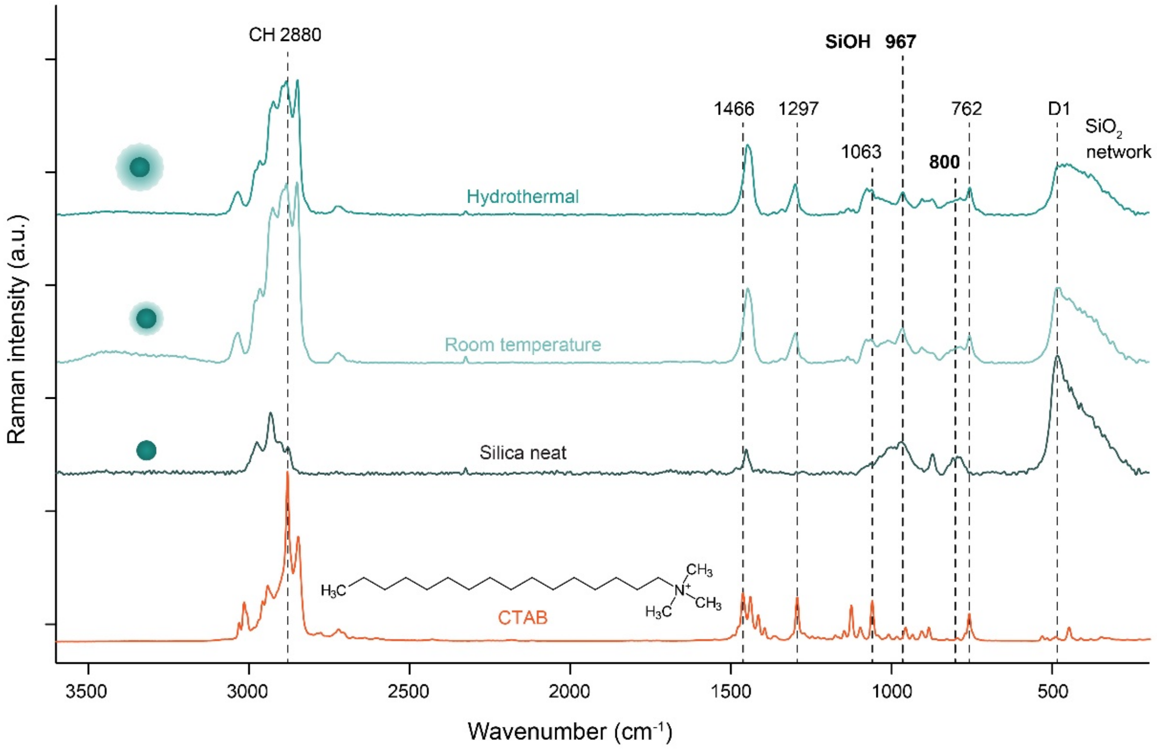

Figure S5. Raman spectra of the bulk nanoparticles synthesized on silica templates before sintering. The dark cyan spectrum shows the neat silica that was used as the core template. The orange spectrum is obtained from the CTAB reference compound.

Figure S6 shows only the characteristic bands (2D, G, and D) of the carbon nanotubes [1], which limits the further analysis of the silica shell.

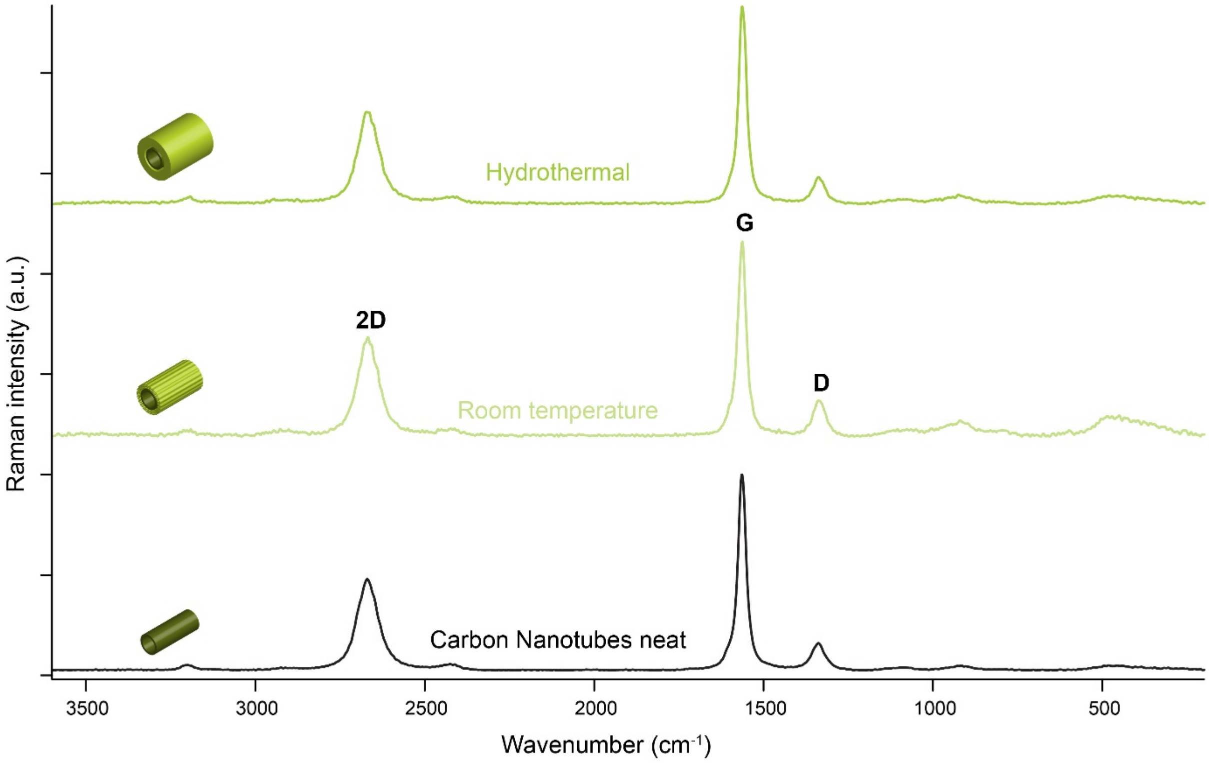

Figure S6. Raman spectra of the bulk nanorods synthesized on carbon nanotubes. The black spectrum shows the reference spectrum of the neat carbon nanotubes which were used as the core template.

References

1. Dresselhaus, M.S.; Dresselhaus, G.; Saito, R.; Jorio, A. Raman spectroscopy of carbon nanotubes. *Phys. Rep.* **2005**, *409*, 47–99, https://doi.org/10.1016/j.physrep.2004.10.006.
